# Photocatalytic and Antibacterial Properties of Doped TiO_2_ Nanopowders Synthesized by Sol−Gel Method

**DOI:** 10.3390/gels8100673

**Published:** 2022-10-20

**Authors:** Silviu Preda, Jeanina Pandele-Cușu, Simona Viorica Petrescu, Elena Mădălina Ciobanu, Gabriela Petcu, Daniela C. Culiță, Nicoleta G. Apostol, Ruxandra M. Costescu, Iuliana Raut, Mariana Constantin, Luminița Predoană

**Affiliations:** 1 Institute of Physical Chemistry “Ilie Murgulescu” of the Romanian Academy, 202 Splaiul Independenței, 060021 Bucharest, Romania; 2 National Institute of Materials Physics, Atomiștilor 405A, 077125 Măgurele, Romania; 3 National Institute for Research & Development in Chemistry & Petrochemistry−ICECHIM, 202 Splaiul Independenței, 060021 Bucharest, Romania; 4 Faculty of Pharmacy, “Titu Maiorescu” University, 16 Gh. Sincai, 040441 Bucharest, Romania

**Keywords:** copper/zinc doped TiO_2_ powders, sol−gel method, thermal behaviour, photocatalytic activity, antibacterial properties

## Abstract

For environmental applications, nanosized TiO_2_-based materials are known as the most important photocatalyst and are intensively studied for their advantages such as their higher activity, lower price, and chemical and photoresist properties. Zn or Cu doped TiO_2_ nanoparticles with anatase crystalline structure were synthesized by sol−gel process. Titanium (IV) butoxide was used as a TiO_2_ precursor, with parental alcohol as a solvent, and a hydrolysing agent (ammonia-containing water) was added to obtain a solution with pH 10. The gels were characterized by TG/DTA analysis, SEM, and XPS. Based on TG/DTA results, the temperature of 500 °C was chosen for processing the powders in air. The structure of the samples thermally treated at 500 °C was analysed by XRD and the patterns show crystallization in a single phase of TiO_2_ (anatase). The surface of the samples and the oxidation states was investigated by XPS, confirming the presence of Ti, O, Zn and Cu. The antibacterial activity of the nanoparticle powder samples was verified using the gram−positive bacterium *Staphylococcus aureus*. The photocatalytic efficiency of the doped TiO_2_ nanopowders for degradation of methyl orange (MO) is here examined in order to evaluate the potential applications of these materials for environmental remediation.

## 1. Introduction

The intensive use of oxide nanoparticles in various applications is based on a number of exceptional properties of these oxides, among which we can mention good thermal and chemical stability, electrical and optical properties and high photocatalytic activity, low cost, etc. Based on potential technological applications, metal oxide nanoparticles are attracting considerable interest from researchers in the fields of materials chemistry, biomedical, optical, electronics, medicine, agriculture, information technology, catalysis, environment, energy, and sensing.

Among these metal oxides, TiO_2_ is one of the most studied materials for many potential applications, particularly for its photocatalytic activity.

The improvement of the photocatalytic activity (by increasing the photon absorption, increasing the recombination time of the electron–hole pairs and improving the separation efficiency of photocarriers) of titanium dioxide was done by self-doping, non-metal doping, transitional metal doping and rare-earth metal doping [1,2,3,4,5,6,7].

**Figure 1:**
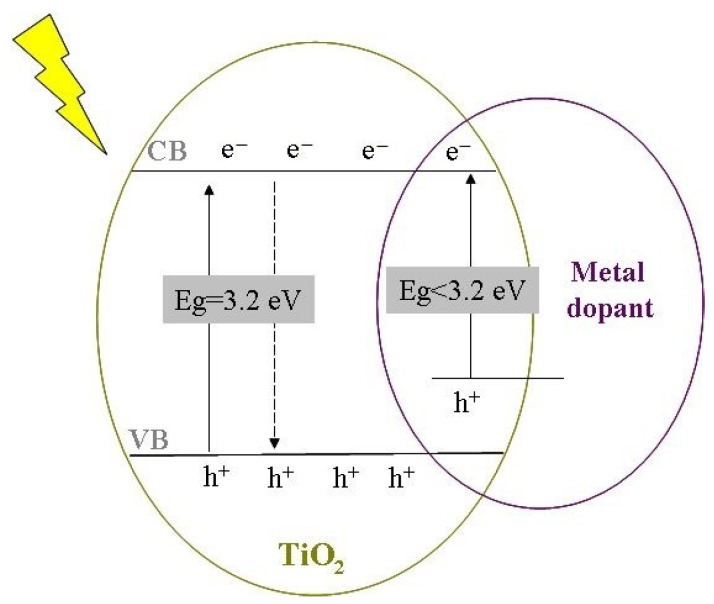
Schematic representation of bandgap narrowing by TiO_2_ doping.

Some of these dopants, such as transitional metals (e.g., Fe, Cr, Ru, Cu, V etc) or non-metals (C, N, S etc.) [8,9,10,11,12,13,14,15] lead to an increase in photocatalytic activity due to the formation of a new electron energy levels inside the bandgap (Figure 1).

The synthesis methods, the doping techniques, and the dopant types influence the structural properties of TiO_2_. Many works reported that a co-catalyst bonded to a semiconductor’s surface can act as an electron trap to reduce the recombination of the photogenerated electron−hole pairs and thus improve the photocatalytic activity [16,17,18]. Furthermore, the loading of metal dopants on TiO_2_ was shown to lead to a red shift, moving the absorption maxima to the visible region, as the wide band of bare TiO_2_ is not suitable for absorbing visible light for practical applications [19,20,21]. However, by increasing thermal treatment the dopants diffuse on the particle’s surface, where they precipitate and create *q*-sites with plasmonic effects, improving the photocatalytic activity of the material [22].

**Figure 2:**
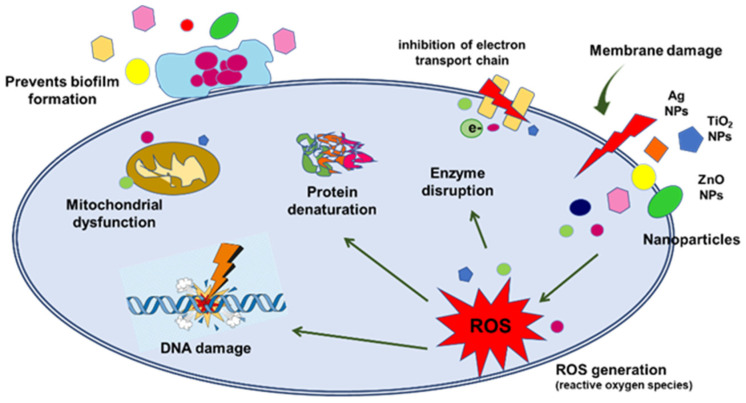
Antimicrobial mechanisms of various nanoparticles (adapted from ref. [25]).

To improve the TiO_2_ antibacterial properties, Cu and Zn doping are an attractive approach because both are antibacterial elements [23,24]. The effect of dopants on the antibacterial properties of titanium dioxide materials was investigated by different groups [10,20,23] and showed that the doped TiO_2_ extend the light absorption spectrum toward the visible light domain and improves the bacteria inactivation efficiency of the nanoparticles. A schematic diagram of the various nanoparticles’ effects on bacteria cells is shown in Figure 2. NPs can attack and kill bacterial cells through multiple mechanisms. Thus, the formation of ROS causes membrane and DNA damage, protein denaturation, inhibiting the electron transport chain, enzyme disruption, and photocatalysis.

For the preparation of TiO_2_ or doped TiO_2_ materials, different methods are available [26,27,28,29,30]. Among them, the sol−gel method is often used to synthesize doped TiO_2_ because it has advantages such as high purity, relatively low processing temperature and the fact that it offers the possibility of controlling stoichiometry [31,32,33,34,35,36].

In accordance with one of the most exhaustive definitions [37], the sol−gel process takes place in two steps: the first step is the formation of an inorganic polymeric network by reaction in solution at low temperatures. During the second step, a conversion of the inorganic amorphous polymers to glass or into crystalline materials takes place. The process temperatures are significantly lower than the melting point of the corresponding oxides.

The advantages of the sol−gel method against other preparation methods are a good mixture of the reagents at the molecular level, leading to a homogeneous end−product; the ability to obtain pre−established structures and shapes (gels, films, fibres, powders, etc.) by varying the experimental conditions [38].

In the present paper, Cu− and Zn− doped TiO_2_ nanometre-sized powders were prepared by the sol−gel method in a basic medium. The aim of this work was the doping of TiO_2_ in order to improve the absorption capacity in a visible light domain to decrease the bandgap and to increase the recombination time by introducing additional energy levels. The influence of the dopant on the structure and the properties of these materials were evaluated. The photocatalytic activity of the doped TiO_2_ nanopowders for degradation of methyl orange (MO) is here examined in order to evaluate the potential of these materials for environmental applications. The antibacterial activity of the nanoparticle powder samples was tested using the gram−positive bacterium *Staphylococcus aureus*.

## 2. Results and Discussion

The samples were investigated following the thermal behaviour, morphology, structure and their properties (antimicrobial or photocatalytic). In the case of the TiO_2_−Cu 2.0% sample a white−green amorphous powder was obtained, while for the TiO_2_−Zn 2.0% sample the obtained powder was amorphous and had a white colour powder.

### 2.1. As Prepared Samples

#### 2.1.1. SEM Results

**Figure 3:**
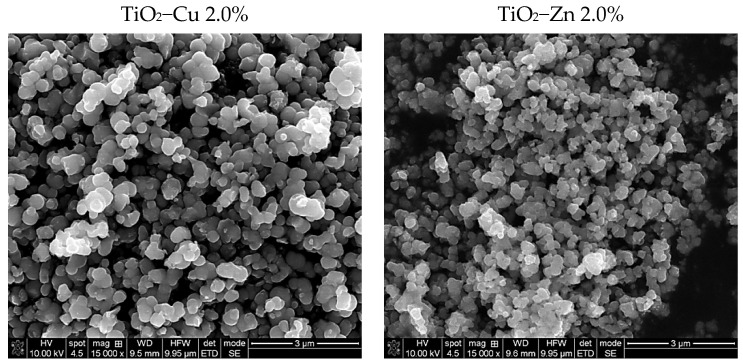
SEM micrographs of the as−prepared samples.

SEM analysis was performed to investigate the morphology of the as-prepared samples, and the micrographs are shown in Figure 3.

According to SEM observations, for the as-prepared samples, the nanoparticles’ surfaces appear to be well-defined with quasi-spherical profiles. The mean diameter, found by Image J software, is 86 nm in TiO_2_−Cu 2.0% sample slightly larger than 75 nm for TiO_2_−Zn 2.0%, which also seems to be more agglomerated.

#### 2.1.2. Thermal Behaviour

**Figure 4:**
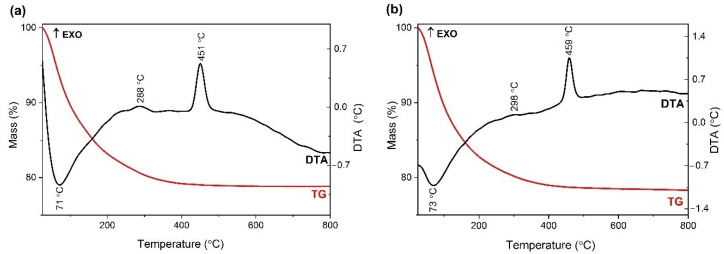
The TG (red)/DTA (black) curves of (**a**) TiO_2_−Cu 2.0% sample and (**b**) TiO_2_−Zn 2.0% sample; heating rate was 10 °C/min and using air as carrier gas.

The thermogravimetric and differential thermal analysis (TG/DTA) were used to examine the thermal behaviour of the as-prepared samples and to set the subsequent thermal treatment. The mass loss and the thermal effects are shown in Figure 4. From Figure 4a the TG curves of the TiO_2_−Cu 2.0% sample show a total mass loss of 21%. Three important heat effects are observed on the DTA curve. The endothermic effect at 71 °C is assigned to the elimination of adsorbed water; the two exothermic effects are the one at 288 °C assigned to the decomposition of the samples, by the elimination of the organic residues and the structural hydroxyls (dehydroxylation), and the one at 451 °C, which can be attributed to the crystallization of anatase phase. In Figure 4b, the TG curves of the TiO_2_−Zn 2.0% sample show a total mass loss of 22.3%.

Three important heat effects are observed on the DTA curve. The endothermic effect at 73 °C is attributed to the elimination of adsorbed water; the two exothermic effects are the one at 298 °C assigned to the decomposition of the samples, by the elimination of the organic residues and the structural hydroxyls (dehydroxylation) and the one at 459 °C, which can be assigned to the crystallization of anatase phase. In both cases, no further weight loss could be observed at temperatures above 500 °C.

#### 2.1.3. XPS on the As-Prepared Samples

**Figure 5:**
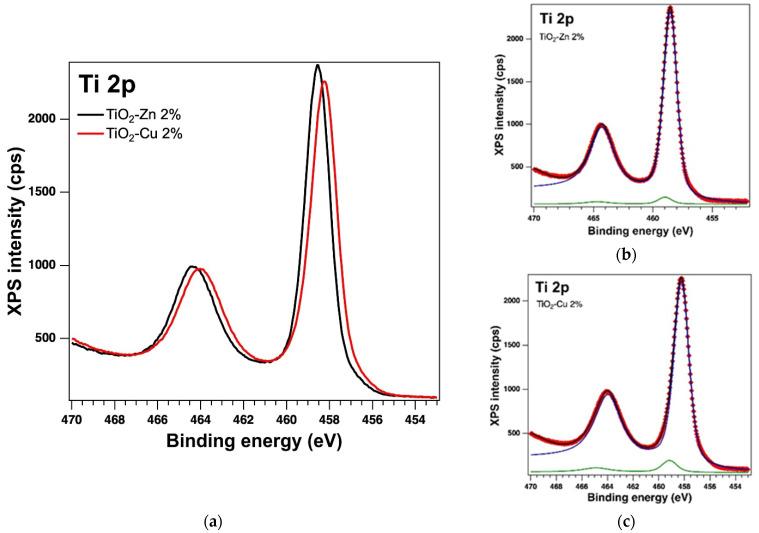
XPS spectra of the core level Ti 2p (**a**) overlayed XPS spectra of the samples prepared by the sol−gel method and the experimental data (red symbols) with the fit and deconvolutions given separately for (**b**) the sample with Zn and (**c**) the sample with Cu; deconvolutions: components C1–blue line and C2–green line.

**Figure 6:**
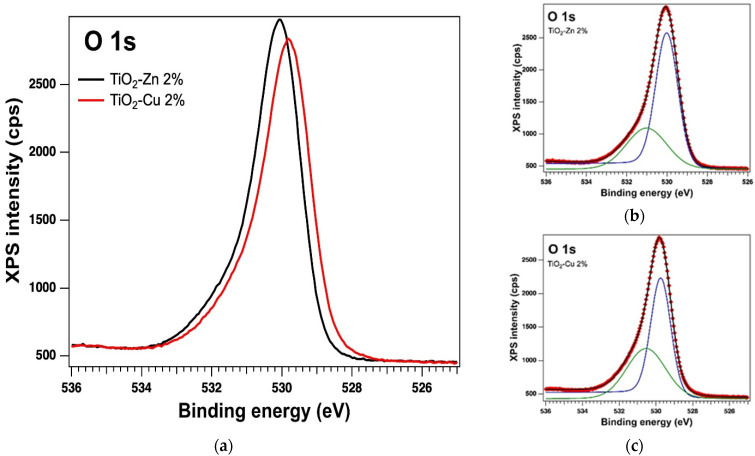
XPS spectra of the core level O 1s (**a**) overlayed XPS spectra of the samples prepared by the sol−gel method and the experimental data (red symbols) with the fit and deconvolutions given separately for (**b**) the sample with Zn and (**c**) the sample with Cu; deconvolutions: components C1–blue line and C2–green line.

**Figure 7:**
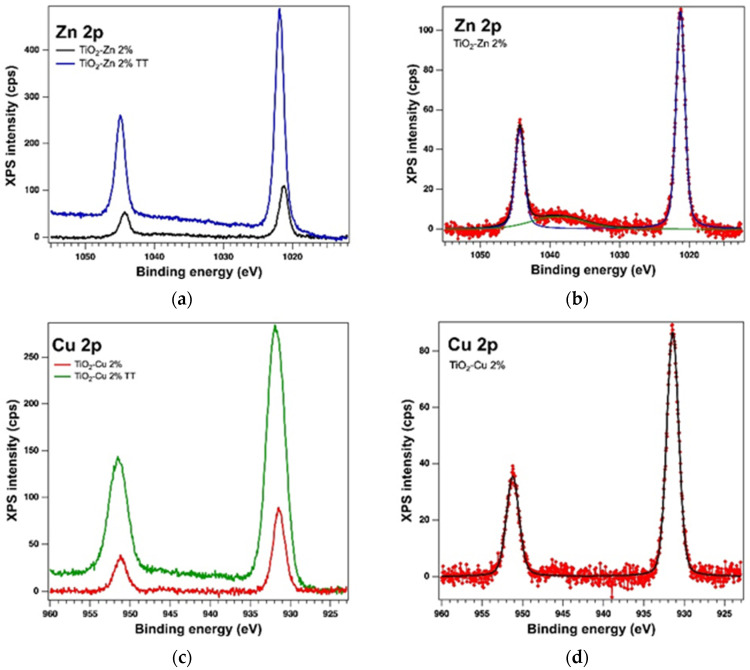
XPS spectra of the core levels Zn 2p (**a**) and Cu 2p (**c**) overlayed XPS spectra of both kinds of samples and the experimental data (red symbols) with the fit and deconvolutions for (**b**) the sol−gel sample with Zn (deconvolutions: components C1–blue line and C2–green line) and (**d**) the sol−gel sample with Cu (only one component–black line).

The oxidation states of the elements and the surface of the samples were analysed by X−ray Photoelectron Spectroscopy (XPS). The analysis showed the presence of the Ti 2p, O 1s, Zn 2p and Cu 2p core levels and indicates the presence of different species as follows: Ti(IV), Zn(II) and Cu(I). The binding energy (BE) for Ti 2p3/2 is around 458.53 eV with a spin-orbit splitting of 5.7 eV, consistent with the database [39]. The Zn 2p3/2 peak is centred at 1021.26 eV attributed to Zn(II) [40] and for Cu 2p3/2 we found the BE at 931.44 eV, which could be attributed to Cu(I), with a very weak specific satellite at about 945 eV [41]. Table 1 lists the essential parameters (binding energy and at. %) obtained as a result of the deconvolutions of the spectra for the core levels of interest, of Ti 2p, O 1s, Zn 2p, and Cu 2p for the as-prepared samples (see the Figure 5, Figure 6 and Figure 7). The values of the amplitude were normalized by the atomic sensitivity factors given by ref. [42].

The as-prepared powders were thermally treated at 500 °C for 1 h. No mass loss was measured above this temperature and anatase phase crystallization is already completed.

### 2.2. Thermally Treated Samples (Powders)

#### 2.2.1. SEM Results

**Figure 8:**
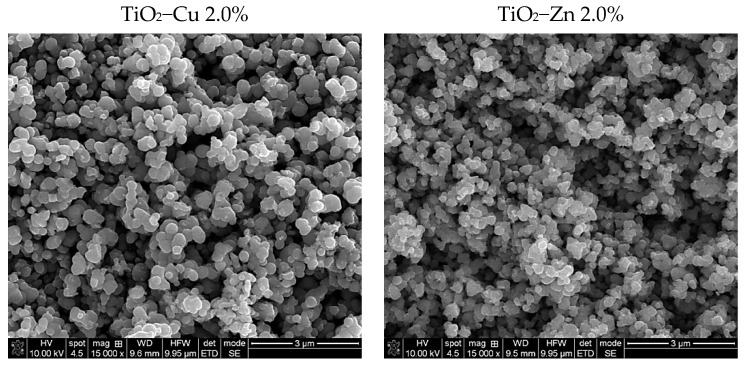
SEM images of the thermally treated samples at 500 °C 1h.

The surface morphology of the thermally treated powders, performed by SEM, is presented in Figure 8, showing nanoparticles clusters with similar sizes compared with as−prepared samples. Following the thermal treatment, the samples’ surfaces are slightly aggregate, with more irregular shapes, related to untreated ones, as confirmed by BET measurements.

#### 2.2.2. X−ray Diffraction (XRD)

**Figure 9:**
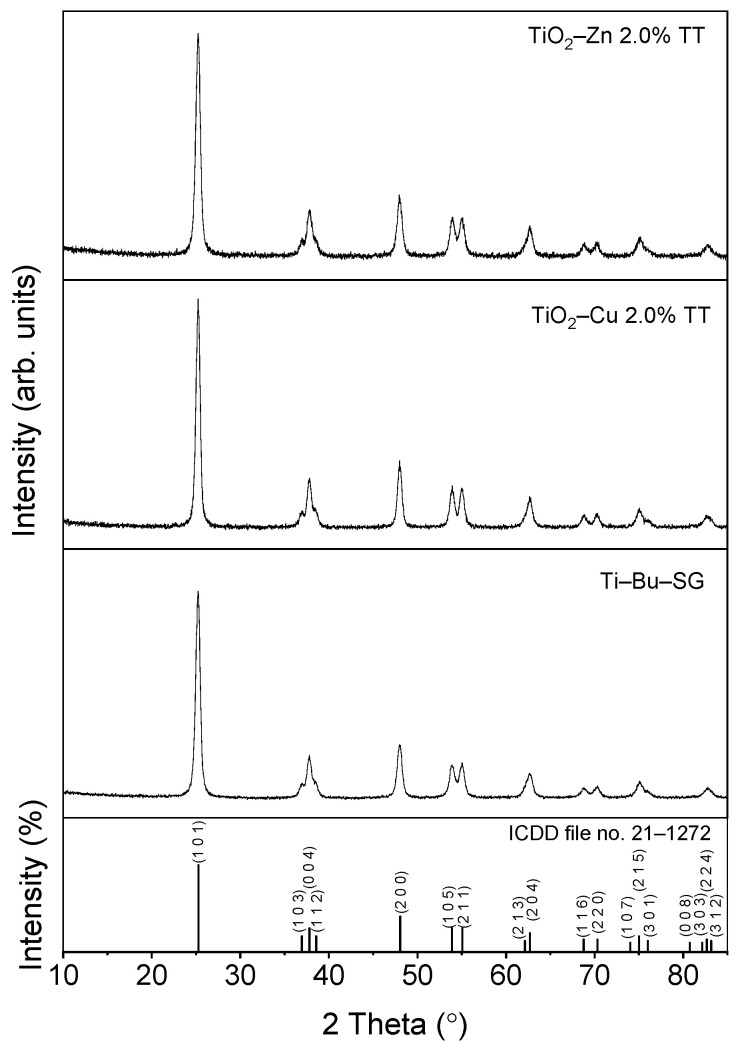
X−ray patterns for powders thermally treated at 500 °C 1 h.

The crystallinity and structure of the thermally treated samples were assessed by X−ray diffraction (XRD). Figure 9 shows the patterns of thermally treated Cu− and Zn−doped TiO_2_ samples at 500 °C for 1 h. Moreover, the undoped TiO_2_ sample (Ti−Bu−SG) was measured as a reference. Single-phase anatase TiO_2_ was identified in all the samples, according to ICDD file no. 21-1272. No diffraction lines related to TiO_2_ polymorph phases or additional diffraction lines related to Zn or Cu compounds were detected in the XRD patterns, which may suggest that Cu and Zn were in a dispersed state or the ions were properly entered into the anatase lattice. The lattice parameters, as well as the mean crystalline domain sizes, calculated by the Williamson−Hall method, are shown in Table 2. No major differences in terms of unit cell parameters are noticed against the standard reference file (ICDD 21-1272), pointing out that Cu and Zn dopants probably substitute for Ti in the TiO_2_ host lattice. The mean crystalline domain sizes of the Ti−Bu−SG and TiO_2_−Cu 2.0% TT samples are similar (~14 nm), while the value for the TiO_2_−Zn 2.0% TT sample is 1 nm smaller (~13 nm).

#### 2.2.3. X−ray Fluorescence (XRF)

Concerning the presence of the dopant in the samples, X−ray fluorescence analysis was performed, which demonstrated the presence of dopant (copper or zinc) in both studied samples. The elemental composition of the samples is listed in Table 3. Other elements (C, S, Si) were detected as traces. Oxide composition by mass percentage was also calculated. More Cu was detected in the final mixture compared to Zn. A possible explanation can be that, during synthesis, a small amount of compound was lost by washing.

#### 2.2.4. XPS on the Thermally Treated Samples

**Figure 10:**
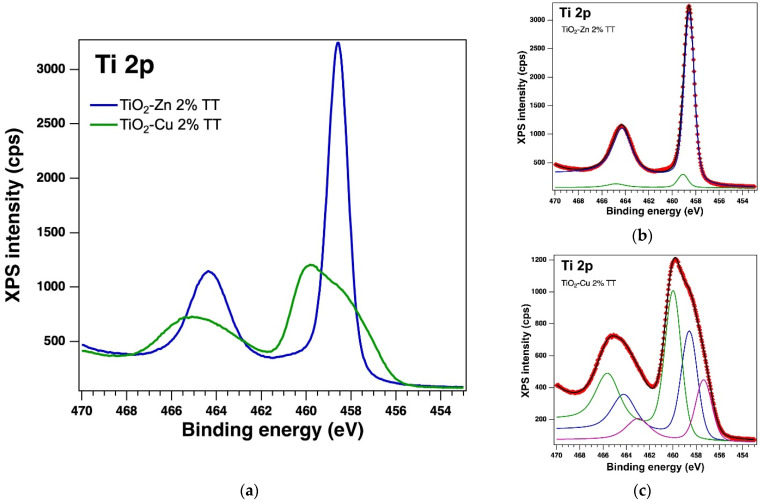
XPS spectra of the core level Ti 2p (**a**) overlayed XPS spectra of the thermally treated samples and the experimental data (red symbols) with the fit and deconvolutions for (**b**) the sample with Zn (deconvolutions: components C1 – blue line and C2–green line) and (**c**) the sample with Cu (deconvolutions: components C1–blue line, C2–green line and C3–magenta line).

**Figure 11:**
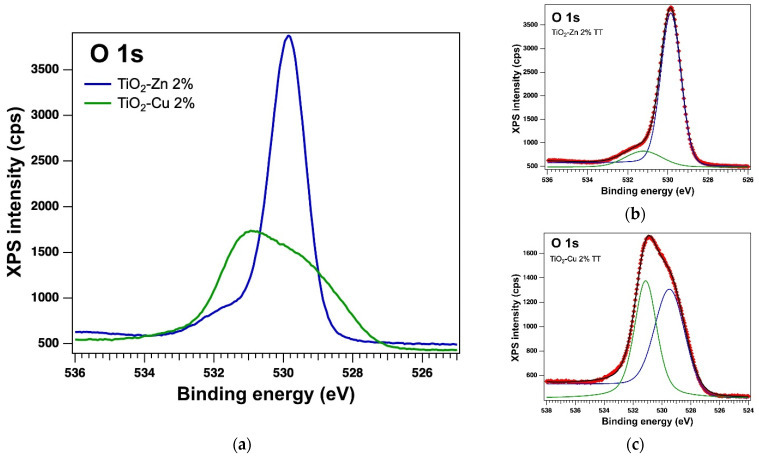
XPS spectra of the core level O 1s (**a**) overlayed XPS spectra of the thermally treated samples and the experimental data (red symbols) with the fit and deconvolutions for (**b**) the sample with Zn and (**c**) the sample with Cu; deconvolutions: components C1–blue line and C2–green line.

**Figure 12:**
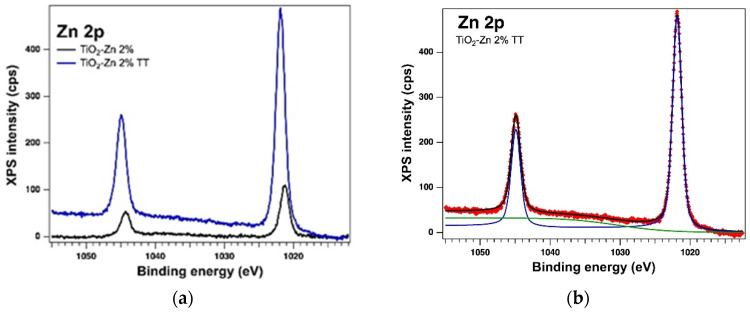

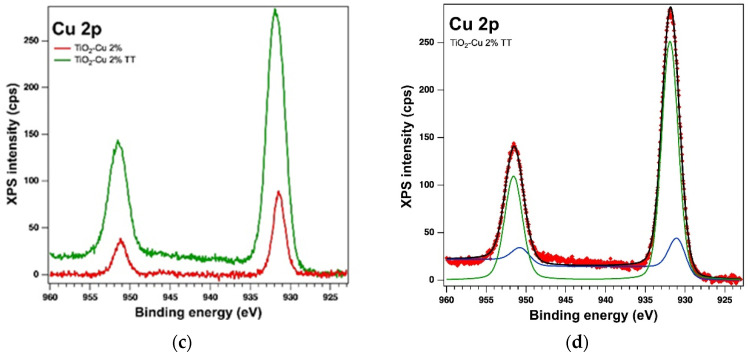
XPS spectra of the core levels Zn 2p (**a**) and Cu 2p (**c**) overlayed XPS spectra of both kinds of samples and the experimental data (red symbols) with the fit and deconvolutions for (**b**) the thermally treated sample with Zn (deconvolution with component C1–blue line; the green line represents an Auger line, which is not taken into account in the chemical analysis) and (**d**) the thermally treated sample with Cu (deconvolutions: components C1–blue line and C2–green line).

The surface of the thermally treated samples was also examined by X−Ray Photoelectron Spectroscopy (XPS). The core levels of interest were deconvoluted using the same method [44] as for the as-prepared samples. In the case of thermally treated samples, the XPS measurements also confirmed the presence of the Ti 2p, O 1s, Zn 2p and Cu 2p core levels and from the deconvolutions, we obtained: Ti(IV), which is the major component, Zn(II) and Cu(I); but in the case of the TiO_2_−Cu 2.0% TT sample we observed the presence of a small amount of Ti(II) and Cu(0), which can be assumed to be related to the thermal treatment. The integral area was used to determine the atomic composition. The integral area was computed using the Voigt profiles deconvolution procedure and scaled the atomic sensitivity factors provided by ref. [42]. The XPS spectra of the Ti 2p, O 1s, Zn 2p and Cu 2p core levels of the thermally treated samples are displayed in Figure 10, Figure 11 and Figure 12. Table 4 lists the binding energy and at. %, obtained as a result of the deconvolutions of the spectra for the core levels of interest.

#### 2.2.5. Antibacterial Activity

The antibacterial activity of the nanoparticle powder samples was investigated using the gram-positive bacterium *Staphylococcus aureus*. In previous experiments using the diffusion method on doped samples, the best results were obtained for *S. aureus* [45], therefore we continued with this bacterium for the experiments in a broth culture medium. For this experiment, the method of testing in a liquid culture medium was used. The evaluation of the antibacterial activity was performed by measuring the absorbance (see Table 5) after 24 h of incubation. The negative control was represented by the nanoparticle powders immersed separately in the culture medium without inoculating the *S. aureus* strain. The real OD (optical density) of the samples is obtained through the subtraction of the OD of the samples with *S. aureus* and the OD of the negative controls (samples containing the nanopowders but without bacteria) and, in the end, the results are compared with the biological positive control of *S. aureus*.

Additionally, the bacterial growth inhibition rate was calculated according to the formula and can be seen in Table 5.

The antibacterial activity of nanopowders against *S. aureus* in Mueller Hinton broth was the highest for the Ti−Bu−SG sample with an inhibition rate of 96.29%, followed by TiO_2_−Cu 2.0% TT and TiO_2_−Zn 2.0% TT, with inhibition of 85.47% and 84.85%, respectively. Many studies have shown the good antimicrobial activity of TiO_2_ nanoparticles [46,47].

#### 2.2.6. Textural Characterization

**Figure 13:**
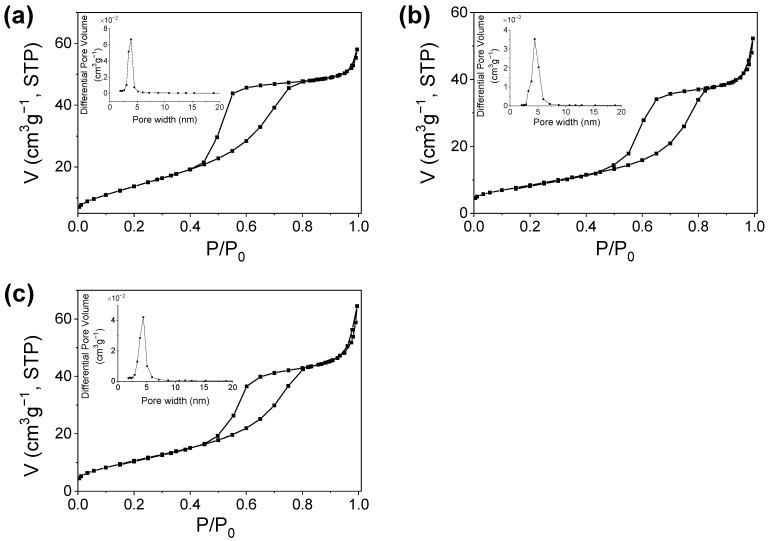
N_2_ adsorption–desorption isotherms and pore size distributions (inset of the figures) of Ti−Bu−SG (**a**), TiO_2_−Cu 2.0% TT (**b**), and TiO_2_−Zn 2.0% TT (**c**) samples.

For the textural characterization of the thermally treated Cu and Zn doped and undoped TiO_2_, the adsorption−desorption isotherms N_2_ were registered and are shown in Figure 13. The specific surface area (SSA) was calculated by the Brunauer−Emmett−Teller (BET) method. The pore size distribution and average pore diameter were determined by Barrett−Joyner−Halenda (BJH) analysis. The textural parameters (BET surface area (SBET), total pore volume, and average pore size) are listed in Table 6. All three samples exhibit type IV isotherms accompanied by H3 hysteresis loops according to IUPAC classification [48], characteristic of mesoporous materials with slit−shape pores. The undoped TiO_2_ sample (Ti−Bu−SG) presents the largest surface area of 52.3 m^2^/g. Doping with Cu and Zn causes a decrease in surface area by 40% and 20%, respectively. At the same time, an increase in average pore diameter and a slight widening of pore size distribution could be observed, most probably due to the higher particle agglomeration. The total pore volume is influenced differently depending on the dopant ion. Compared to undoped TiO_2_, it shows a contraction of 10% upon doping with Cu and an increase of 10% upon doping with Zn, respectively.

#### 2.2.7. Photocatalysis Investigation

**Figure 14:**
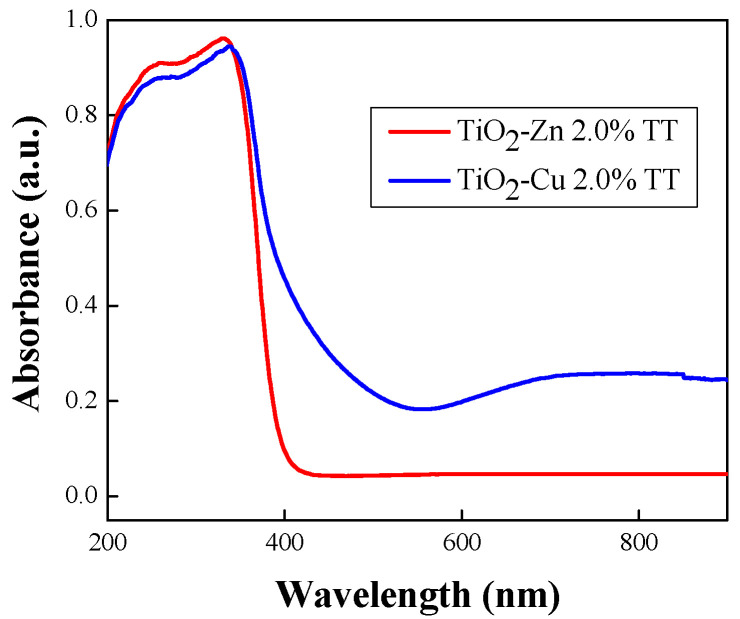
DR−UV−Vis absorption spectrum for TiO_2_−Zn 2.0% TT and TiO_2_−Cu 2.0% TT samples.

Figure 14 shows the absorbance spectra of TiO_2_−type materials, undoped and doped with 2.0% Zn or Cu, respectively. The spectrum of undoped TiO_2_ (Ti−Bu−SG sample) shows a band edge absorption located at ~370 nm, typically for the band gap of TiO_2_ nanoparticles in the anatase phase. In the case of sample TiO_2_−Zn 2.0% TT, no additional absorption band can be observed, ZnO exhibiting a band at ~369 nm [49]. Furthermore, a decrease in the absorption intensity after Zn addition can be observed, probably due to the displacement of titanium ions by zinc into the anatase phase, as XRD analysis suggested. By contrast, the absorption bands of TiO_2_−Cu 2.0% at around 250–550 nm can be observed due to the O^2−^ (2p) → Ti^4+^ (3d) transitions in the tetrahedral symmetry. Additionally, a broad absorption band in the visible domain was evidenced, proving the presence of Cu species. According to Colon et al. [50], the absorption band between 400 nm and 600 nm, can be assigned to the presence of Cu^1+^ clusters, resulting after a partial reduction of Cu^2+^ species by a strong interaction between TiO_2_ support and copper nitrate. This strong interaction is also suggested by the obtaining of Ti(II) species in the case of the TiO_2_−Cu 2.0% sample, as XPS results revealed. The large absorption band located between 600–800 nm indicates the increase of CuO in octahedral symmetry [50]. Thus, the introduction of the dopants leads to a red shift of the absorption band, as was previously reported [51]. This behaviour can be attributed to the formation of impurity levels within the band gap states of TiO_2_. The band gap value of the bare TiO_2_ was 3.15 eV. When doping with a transitional metal (Cu or Zn), the band gap values decrease (see Table 6), these values being in agreement with the previously reported data [51,52]. It was suggested through theoretical calculations that the band gap narrows after Cu doping due to new electronic levels in the valence band resulting from the covalent interaction between Cu and O [53]. For the TiO_2_−Zn 2.0% TT sample, the band gap energy decreased due to the synergistic effect between the conduction band of TiO_2_ and that of ZnO. Both impurities and defects were introduced to the forbidden band of TiO_2_, leading to the formation of some sub-bands, thus, the value of the band gap energy was decreased [52].

**Figure 15:**
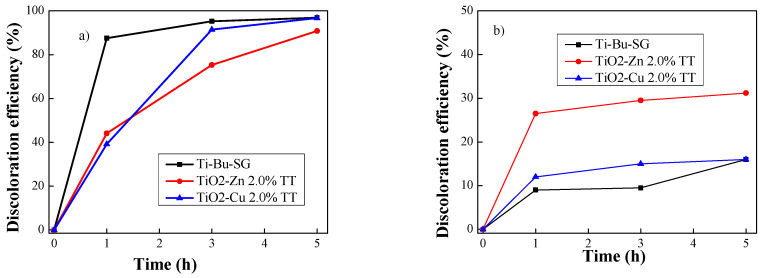
Discoloration of methyl orange under UV (**a**) and visible (**b**) light irradiation using synthesized materials.

The photocatalytic degradation of methyl orange solution in the presence of undoped and doped TiO_2_ was carried out under UV and visible light irradiation. The results presented in Figure 15 showed that the introduction of a doping element in titanium dioxide leads to a higher photocatalytic activity.

It was observed that in the UV domain, the TiO_2_−Cu 2.0% TT sample, with crystalline domains size of 14 nm, presented the highest activity in photocatalytic degradation of MO, removing ~97% of the dye in 300 min (Figure 15), while the sample with a smaller value of crystalline domains size (13 nm), TiO_2_−Zn 2.0% TT, presented a lower degradation efficiency, about 90%. In the case of visible light irradiation, the most effective was the sample TiO_2_−Zn 2.0% TT, removing a total of 30% of MO in 300 min, while the sample doped with Cu presents activity comparable with the bare TiO_2_ (Ti−Bu−SG sample) (~ 16%). It can be observed that the presence of a doping metal (Cu, respectively, Zn) led to an increase of photocatalytic activity in the visible domain compared to bare TiO_2_. Furthermore, the higher photocatalytic activity of the TiO_2_−Zn 2.0% TT materials can be attributed to their increased absorption capacity due to a higher surface area compared to TiO_2_−Cu 2.0% TT (see Table 6) a process that ensures the presence of the dye molecules close to the active centres located on the surface, thus facilitating the photodegradation process.

## 3. Conclusions

In the present work, copper- and zinc-doped TiO_2_ materials prepared by the sol–gel method were investigated for their photocatalytic and antibacterial activities. The main results can be summarized as follows: by thermal analysis, the heat treatment temperature was set to 500 °C in order to obtain a crystalline phase without organic residues. By XRD, single-phase anatase with nanometre-sized crystallites was identified and, by XRF, the presence of the dopants was detected. The surface and the oxidation states of the samples prepared by the sol−gel method and thermally treatment method investigated by XPS confirmed, also, the presence of Ti 2p, O 1s, Zn 2p and Cu 2p core levels. The textural characterization of the TiO_2_ doped with Cu or Zn causes a decrease in surface area by 40% and 20%, respectively. Compared to undoped TiO_2_, it shows a contraction of 10% upon doping with Cu and an increase of 10% upon doping with Zn, respectively. The photocatalytic activity of doped TiO_2_ increased under visible light irradiation compared to the undoped TiO_2_ due to the newly formed sub-bands and to the active centres located on the surface, thus facilitating the photodegradation process. In the UV domain, a relation between the crystalline domain sizes and the photocatalytic discoloration of methyl orange was noticed. This paper examined the inhibitory effect determined by TiO**_2_** nanopowders against *S. aureus*. The results of the antimicrobial test confirmed a considerable antimicrobial activity against gram-positive bacteria of the doped nanopowders. 

## 4. Materials and Methods

### 4.1. Sample Preparation

**Figure 16:**
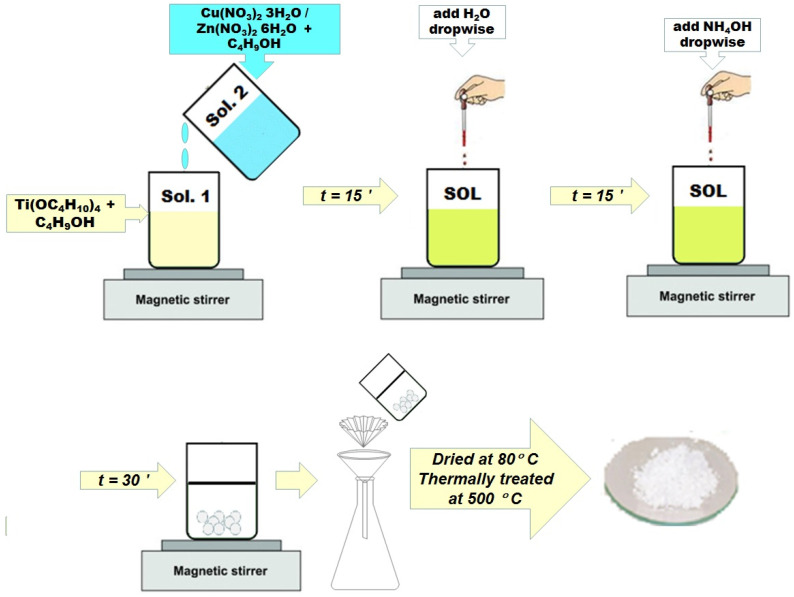
The flowchart of the sample preparation by sol-gel method.

Nanopowders of Cu− or Zn−doped TiO_2_ were prepared by the sol−gel method. The initial calculated compositions corresponding to a TiO_2_:CuO or TiO_2_:ZnO molar percentage of 98:2 were chosen. The reaction started from titanium (IV) butoxide [TBOT = Ti(OC_4_H_10_)_4_] (Merck) as a precursor of TiO_2_ and copper nitrate [Cu(NO_3_)_2_·3H_2_O] (Merck) as a precursor of CuO or zinc nitrate [Zn(NO_3_)_2_·6H_2_O] (Merck) as a precursor of ZnO. Butanol [C_4_H_9_-OH] (J.T. Baker) was used as the solvent, H_2_O as the hydrolysis agent, and ammonium hydroxide [NH_4_OH] (Riedel-de Haën) as the catalyst. The synthesis took place at room temperature and the solution of reagents was homogenized for 60 min. The oxide powder was separated by filtration from the solution, washed with distilled water to remove adsorbed compounds, dried and then thermally treated at 500 °C, in air, with a plateau of 1 h and a heating rate of 1 °C/min, in order to eliminate the water and organic residues and to obtain crystallized nanometre-sized powders. A flowchart of the methodology used for the sample preparation is given in Figure 16. The thermal treatment was established based on the TG/DTG/DTA results.

The samples were denoted (TiO_2_−Cu 2.0%) and (TiO_2_−Zn 2.0%), and the thermally treated samples (TiO_2_−Cu 2.0% TT) and (TiO_2_−Zn 2.0% TT), respectively. 

The composition of the solutions and the experimental conditions used are shown in Table 7.

The synthesis procedure for the sample Ti−Bu−SG was described in our previous work (ref. [43]).

### 4.2. Methods of Characterization

The thermal behaviour of the as-prepared samples was determined by thermogravimetric and differential thermal analysis (TG/DTA) using Mettler Toledo TGA/SDTA 851e (Greifensee, Switzerland) equipment in open Al_2_O_3_ crucibles and in flowing air atmosphere. The maximum temperature was set at 800 °C and the heating rate was 10 °C/min. 

SEM micrographics were recorded using FEI Quanta 3D FEG microscope (FEI, Brno, Czech Republic), operating at an accelerating voltage of 10 kV. The specimens were placed on conductive carbon tape and scanned in high vacuum mode in an uncoated state.

X−ray Photoelectron Spectroscopy (XPS) was performed in an AXIS Ultra DLD (Kratos Surface Analysis, Manchester, UK) system, using Al K_α1_ (1486.74 eV) radiation produced by a monochromatized X−ray source at operating power of 240 W (12 kV × 20 mA) and a charge neutralizer operating at 1.7 A filament current, 2.8 V charge balance, 2.00 V filament bias. High-resolution core-level spectra were recorded using hybrid lens mode, 40 eV pass energy, and slot aperture. The binding energy scale was calibrated to the C 1s standard value of 284.6 eV. The core level spectra were deconvoluted using Voigt profiles, based on the methods described in ref. [44].

X−Ray Diffraction (XRD) patterns were recorded using a Rigaku Ultima IV X−ray diffractometer. The equipment was set in parallel beam geometry, with cross beam optics (CBO), operated at 40 kV and 30 mA, using CuKα radiation. The data were collected over the 2θ range 10−80° at a scanning rate of 2°/min. Rigaku’s PDXL software, connected to ICDD PDF−2 database, was used for phase identification. Lattice constants were refined using the diffraction line position. The mean crystalline domain size is calculated from the diffraction line width. The diffraction line position and width were corrected by the external standard method.

X−ray fluorescence (XRF) was used for elemental analysis. The measurements were performed using a Rigaku ZSX Primus II spectrometer (Rigaku Corp., Tokyo, Japan), equipped with 4.0 kW X−ray Rh tube. EZ−scan combined with Rigaku SQX fundamental parameters software (standard less) was used for data analysis.

Gram−positive bacteria *Staphylococcus aureus* ATCC 25,923, purchased from the German Collection of Microorganisms and Cell Cultures (DSMZ) (Braunschweig, Germany), was used in the experiment. The working method for testing antibacterial activity of nanoparticle powders was as follows. First, the bacterial strain *Staphylococcus aureus* was replicated on the TSA (Tryptic Soy Agar) medium and incubated at 35 ± 2 °C for 24 h. The working inoculum was made by suspending 2–3 bacterial colonies in sterile physiological water (0.85%), obtaining turbidity of 3 × 10^5^ CFU/mL, which was adjusted spectrophotometrically by measuring the absorbance. The study was carried out in 100-millilitre Erlenmeyer flasks with 30 mL culture medium. Doped TiO_2_ nanopowders were tested using a concentration of 200 µg mL^−1^. The nanoparticle powders were immersed in the Mueller Hinton broth (MHB) inoculated with 1 mL suspension of *Staphylococcus aureus* and incubated at 35 ± 2 °C for 24 h and 130 rpm. The composition of the culture media was Mueller Hinton broth from Scharlau, Sentmenat, Spain (1.5 g/L starch; 2 g/L meat extract; 17.5 g/L peptone). Tryptic Soy Agar from Scharlau, Sentmenat, Spain (2.5 g/L dextrose; 5 g/L sodium chloride; 20 g/L peptone; 2.5 g/L dipotassium phosphate; 15 g/L agar). Nanoparticle powders were also immersed in the culture medium without bacterial strain for the negative control. The positive biological control does not contain nanoparticle powders. The samples were inoculated in five replicates in 96-well plates, 200 μL in each well and incubated at 35 ± 2 °C for 24. After incubation, the absorbance was measured at 600 nm with the help of the Clariostar plate reader.

The inhibition rate (%) was determined using the following formula [54]:

(1) $$\mathrm{Inhibition}\;\mathrm{of}\;\mathrm{Efficiency}\;\left(\%\right)=\mathrm{Control}\;O.D.\times \frac{\mathrm{Test}\;O.D.}{\mathrm{Control}\;O.D.}\times 100$$

Nitrogen adsorption−desorption isotherms at 77 K were recorded on a Micromeritics ASAP 2020 automated gas adsorption system (Norcross, GA, USA). The samples were degassed at 200 °C for 5 h under vacuum before analysis. 

The UV−Vis diffuse reflectance spectra were recorded on a JASCO V570 spectrophotometer (Tokyo, Japan). Photocatalytic experiments were carried out in batches with 5 mg photocatalyst in 10 mL methyl orange (MO) dye (1 × 10^−5^ M). The suspension was stirred during the experiment. The reaction mixture was first stirred in dark for 30 min in order to establish the adsorption of MO dye on the photocatalyst surface. Then it was irradiated in a closed box with an UV−Vis lamp at specific wavelengths. Photocatalytic experiments were performed for 300 min. There were taken the same aliquots of MO solution at regular time intervals and filtered using a 0.45 mm Millipore film in order to evaluate the progress of the reaction. The discoloration efficiency was evaluated as reported before [55].

## Figures and Tables

**Table 1:** Binding energies (BE) at. % and interpretation of the components of the core levels of interest for the samples prepared by sol-gel method.

Sample	Core Levels		BE (eV)	at. %	Interpretation
TiO_2_−Zn 2.0%	Ti 2p	C1	458.53	42.40	Ti(IV) vol.
		C2	459.00	1.60	Ti(IV) surf.
	O 1s	C1	530.02	35.61	Ti(IV) (vol + surf)
		C2	531.02	19.89	Ti(IV) + Zn(II) + cont
	Zn 2p	C1	1021.26	0.50	Zn(II)
TiO_2_−Cu 2.0%	Ti 2p	C1	458.23	42.35	Ti(IV) vol.
		C2	459.10	2.71	Ti(IV) surf.
	O 1s	C1	529.74	29.93	Ti(IV)
		C2	530.54	24.52	Ti(IV) + Zn(II) + cont
	Cu 2p	C1	931.44	0.48	Cu(I)

**Table 2:** The lattice parameters and the mean crystalline domain sizes (Dm) of the samples.

Sample	*a = b* (Å)	*c* (Å)	D_m_ (nm)
Ti−Bu−SG [43]	3.7837 (6)	9.4969 (15)	14
TiO_2_−Cu 2.0% TT	3.7892 (2)	9.5145 (6)	14
TiO_2_−Zn 2.0% TT	3.7854 (10)	9.496 (3)	13
Anatase, syn ICDD 21−1272	3.7850	9.5140	-

**Table 3:** XRF results of the analysed samples.

Sample	Composition	Values	U.M.	Line
TiO_2_−Zn 2.0%	Ti	58.0482	mass%	Ti−KA
	Zn	1.6157	mass%	Zn−KA
	O	39.2373	mass%	O−KA
	C, S, Si (traces)	1.0989	mass%	
	TiO_2_	94.1104	mass%	Ti−KA
	ZnO	1.9373	mass%	Zn−KA
	C, S, Si oxides (traces)	3.9524	mass%	
	Ti	58.7187	mass%	Ti−KA
TiO_2_−Cu 2.0%	Cu	1.9474	mass%	Cu−KA
	O	38.3098	mass%	O−KA
	C, Si, S (traces)	1.0241	mass%	
	TiO_2_	94.0071	mass%	Ti−KA
	CuO	2.3092	mass%	Cu−KA
	C, S, Si oxides (traces)	3.6837	mass%	

**Table 4:** Binding energies, at. % and interpretation of the components of the core levels of interest for the thermally treated samples.

Sample	Core Levels		BE (eV)	at. (%)	Interpretation
TiO_2_−Zn 2.0% TT	Ti 2p	C1	458.58	0.78	Ti(IV) vol.
		C2	459.78	39.04	Ti(IV) surf.
	O 1s	C1	529.85	48.77	Ti(IV) (vol + surf)
		C2	531.22	9.21	Ti(IV) + Zn(II) + cont
	Zn 2p	C1	1021.85	2.2	Zn(II)
TiO_2_−Cu 2.0% TT	Ti 2p	C1	457.29	6.8	Ti(II)
		C2	458.56	12.42	Ti(IV) vol.
		C3	459.97	18.12	Ti(IV) surf.
	O 1s	C1	529.43	29.96	Ti(IV) (vol + surf)
		C2	531.13	30.19	Cu(I) + cont
	Cu 2p	C1	930.95	0.32	Cu(0)
		C2	931.91	2.19	Cu(I)

**Table 5:** Absorbance values for nanopowders in broth medium.

Sample	Concentration	OD of Sample with *S. aureus*	Inhibition Rate %
Ti−Bu−SG	200 µg mL^−1^	0.060	96.29
TiO_2_−Zn 2.0% TT	200 µg mL^−1^	0.235	85.47
TiO_2_−Cu 2.0% TT	200 µg mL^−1^	0.245	84.85
Biological positive control of *S. aureus*	3 × 10^5^ CFU mL^−1^	1.618	-

**Table 6:** The specific BET surface areas (S_BET_), total pore volume (V_total_), average pore diameter (d) and band gap values of the samples.

Sample	S_BET_ (m^2^g^−1^)	V_total_ (cm^3^g^−1^)	d_BJH_ (nm)	Band Gap (eV)
Ti−Bu−SG	52.3	0.089	4.4	3.15
TiO_2_−Cu 2.0% TT	31.3	0.080	6.2	1.5
TiO_2_−Zn 2.0% TT	41.5	0.099	6.1	2.85

**Table 7:** The composition of the solution and the experimental parameters of the sol preparation.

Sample	Precursors	Molar Ratio			pH Sol	Experimental Conditions	
		$\frac{ROH}{\sum precursor}$	$\frac{H_{2}O}{\sum precursor}$	$\frac{catalyst}{\sum precursor}$		T (°C)	t (h)
TiO_2_−Cu 2.0%	Ti(OC_4_H_10_)_4_ + Cu(NO_3_)_2_·3H_2_O	36.5	3	0.003	10	25	60
TiO_2_−Zn 2.0%	Ti(OC_4_H_10_)_4_ + Zn(NO_3_)_2_·6H_2_O	36.5	3	0.003	10	25	60

ROH = C_4_H_9_-OH.
